# On the analysis of EEG power, frequency and asymmetry in Parkinson’s disease during emotion processing

**DOI:** 10.1186/1744-9081-10-12

**Published:** 2014-04-09

**Authors:** Rajamanickam Yuvaraj, Murugappan Murugappan, Norlinah Mohamed Ibrahim, Mohd Iqbal Omar, Kenneth Sundaraj, Khairiyah Mohamad, Ramaswamy Palaniappan, Edgar Mesquita, Marimuthu Satiyan

**Affiliations:** 1 School of Mechatronic Engineering, Universiti Malaysia Perlis (UniMAP), Arau, Malaysia; 2 Neurology Unit, Department of Medicine, UKM Medical Center, Kuala Lumpur, Malaysia; 3 Faculty of Science and Engineering, University of Wolverhampton, Telford, UK; 4 University of Minho, Braga, Portugal

**Keywords:** Emotion, EEG Power, Frequency bands, Hemispheric asymmetry

## Abstract

**Objective:**

While Parkinson’s disease (PD) has traditionally been described as a movement disorder, there is growing evidence of disruption in emotion information processing associated with the disease. The aim of this study was to investigate whether there are specific electroencephalographic (EEG) characteristics that discriminate PD patients and normal controls during emotion information processing.

**Method:**

EEG recordings from 14 scalp sites were collected from 20 PD patients and 30 age-matched normal controls. Multimodal (audio-visual) stimuli were presented to evoke specific targeted emotional states such as happiness, sadness, fear, anger, surprise and disgust. Absolute and relative power, frequency and asymmetry measures derived from spectrally analyzed EEGs were subjected to repeated ANOVA measures for group comparisons as well as to discriminate function analysis to examine their utility as classification indices. In addition, subjective ratings were obtained for the used emotional stimuli.

**Results:**

Behaviorally, PD patients showed no impairments in emotion recognition as measured by subjective ratings. Compared with normal controls, PD patients evidenced smaller overall relative delta, theta, alpha and beta power, and at bilateral anterior regions smaller absolute theta, alpha, and beta power and higher mean total spectrum frequency across different emotional states. Inter-hemispheric theta, alpha, and beta power asymmetry index differences were noted, with controls exhibiting greater right than left hemisphere activation. Whereas intra-hemispheric alpha power asymmetry reduction was exhibited in patients bilaterally at all regions. Discriminant analysis correctly classified 95.0% of the patients and controls during emotional stimuli.

**Conclusion:**

These distributed spectral powers in different frequency bands might provide meaningful information about emotional processing in PD patients.

## Background

Parkinson’s disease (PD) is a common progressive neurodegenerative disorder of the central nervous system [1]. Nowadays, PD influences a large part of worldwide population. About 1% of the population over 55 years of age is affected by this disease [2]. The motor clinical symptoms of PD such as resting tremor, rigidity, bradykinesia and postural instability [3,4] results from dopaminergic deficiency in the basal ganglia. In addition, PD is also characterized by the presence of non-motor symptoms including disruption in emotion information processing [5], which have been found in over 50% of newly diagnosed PD patients [6] and can appear in any stage of disease progression [7].

Individuals with PD show impairments in the ability to recognize emotions from facial expression [5,8-10], speech prosody [11,12] and show reduced startle reactivity to highly arousing unpleasant pictures [13,14]. There is sparse event related potential (ERP) evidence that early processing of emotional prosody (mismatch negativity [15]) and faces (early posterior negativity [16]) may be affected in PD. While there are also reports of intact emotion recognition [5,16-21], others have documented impairments in recognizing some of the basic emotions (anger, fear, disgust, happiness, sadness, and surprise) but not other emotions [9,22]. Most recently, lateralization (left versus right) of emotion recognition in PD has been debated. For example, Clark et al. reported no asymmetry effects on explicit emotion categorization [8]. Ariatti et al. and Yip et al. reported problems in categorizing disgust prosody in patients with predominantly right-sided [23,24]. While Ventura et al. reported that predominantly left sided patient’s exhibit recognition of sadness emotion [25]. Finally, it is not yet clear whether deficits appear in recognizing emotion only in one stimulus modality (i.e., facial expressions [8]) or more (facial displays and prosody [11,23]; facial displays, voices, and verbs [12]). Altogether, experimental evidence so far supports the view of impairments in emotion processing in PD. Most studies on emotion recognition mentioned above dealt with behavioral responses (i.e., participants were asked to match, to identify, to judge, or to rate the emotional stimuli) whereas very few studies dealt with physiological measures (i.e., startle eye blink and ERPs).

Furthermore, PD is characterized by a loss of dopaminergic innervation of the basal ganglia, including the ventral striatum, and the subthalmic nucleus. These structures are highly interconnected with, for instance the amygdala and the orbifrontal cortex, brain regions associated with emotion recognition [23]. In addition, it is well documented that emotional processing involves a multitude of processes in several brain circuits. One example is the somatic marker hypothesis by Damasio, which states that emotions results from an interpretation of somatic states [26]. Thus, impairments in the processing of emotional information by PD patients are also reflected in the characteristics of electrical activation of the brain i.e., electroencephalogram (EEG). In general, due to their noninvasive recording procedure and temporal resolution, EEG signals have been widely used in order to study brain activity relating to affective responses. Evidence of such activity is reported in the majority of EEG frequency bands i.e., delta (δ: 1 – 4 Hz), theta (θ: 4 – 8 Hz), alpha (α: 8 – 13 Hz), beta (β: 13 – 30 Hz) and gamma (ϒ: 30 – 60 Hz). In line with results from healthy participant EEG emotion study, one of the common indicators of emotional states is the alpha-power asymmetry derived from the spectral differences between a symmetric electrode pair at the anterior areas of the brain [27]. Other spectral changes and brain regions were also reported, which are associated with emotional responses, such as the alpha power changes at right parietal lobe [28], theta power changes at right parietal lobe [29], the frontal midline theta power [30], beta –power asymmetry at the parietal region [31], and the gamma spectral changes at the right parietal regions [32].

This study aims to investigate whether differences in EEG frequency bands, induced by the emotional information could be used to discriminate PD patients and normal controls (NCs). For this purpose, we utilized traditional EEG spectral measures of absolute and relative power as well as the measures of EEG mean frequency. We also studied the functional connectivity between brain regions by examining inter-hemispheric and intra-hemispheric relationships responses to emotional stimuli. Statistical analysis was used to evaluate the extracted features between the two groups. To our knowledge, no study has yet been conducted to explore the correspondence between emotional states and EEG frequency bands in PD patients.

## Materials and methods

### Participants

Twenty three PD patients and 30 NC that have been matched for age, education level, and gender participated in the study. Due to excessive artifacts (body movements and closing of the eyes), three participants of the PD group had to be excluded from the further analysis, resulting in a sample of 20 PD patients (10 men and 10 women) and 30 NC (13 men and 17 women). The PD patients were recruited from the clinic Neurology outpatient service of the Hospital University Kebangsaan Malaysia (HUKM) medical center, Kuala Lumpur, Malaysia. All of them had been diagnosed with idiopathic PD by a neurologist. All patients were optimally medicated during testing session (ON state) with d2-agonist (n = 18); carbidopa/L-dopa (n = 13), monoamine oxidase B (MAO-B) inhibitor (n = 7), catechol-O-methyltransferase (COMT) inhibitor (n = 5), amantadine (n = 5), or anticholinergics (n = 3). The average duration of PD (post-diagnosis) in the group was 5.75 years [standard deviation (SD) = 3.52, range = 1–12 years]. The severity of motor signs in the group could be characterized as mild to moderate; all patients fit Hoehn and Yahr stages (H & Y) [33] I – III (Stage I = unilateral disease with mild symptoms, stage II = bilateral involvement, stage III = bilateral symptoms with postural and gait disturbances) with a mean Unified Parkinson’s Disease Rating Scale (UPDRS) [34] motor score of 17.05 (SD = 3.15). None of the patients had coexisting neurological (e.g., epilepsy) or psychiatric disturbance (e.g., major depression or anxiety, psychotic symptoms, etc.) that might independently influence their cognitive functioning.

The healthy control participants were recruited through the hospital’s medical unit community and/or from patient’s relatives. Exclusion criteria for controls included any psychiatric or neurological disorder. To exclude dementia or depression, participants scoring 24 or lower on the Mini-Mental State Examination (MMSE) [16,35] or 18 or higher on the Beck Depression Inventory (BDI) [15,36] were excluded. All participants were right-handed as determined by self-report and confirmed by Edinburgh Handedness Inventory (EHI) [37]. This test consisted of 10 questions asking for the preferred hand for a series of activities (e.g. writing, throwing, using scissors, etc.). All participants reported normal or corrected-to-normal vision, and intact hearing was formally established in all participants by administering a pure tone audiometric screening of both ears to ensure acceptable normal hearing threshold (minimum 30 dB HL at 0.5, 1, 2, and 4 kHz, for the better hearing). All participants/caretaker gave informed consent before completing the study, which was ethically approved by the Faculty of Medicine, Institutional Review Board of the University Kebangsaan Malaysia. All participants were paid for their participation.

Patients and controls were comparable in demographic variables such as age (PD: M = 59.05 years, SD = 5.64; NC: M = 58.43 years, SD = 3.01; t (48) = 0.502, p = 0.61), gender distribution (PD: 10 men, NC: 13 men; *x*^*2*^ (1, N = 50) = 0.21, p = 0.68), and education level (PD: M = 10.45 years, SD = 4.8; HC: M = 11.02 years, SD = 3.24; t (48) = −0.62, p = 0.51). As shown in Table 1, PD patients did not differ in mean MMSE scores, mean BDI scores, as well as mean EHI scores.

**Table 1:** Demographic and clinical characteristics of PD patients and normal controls

**Variable**	**PD (n = 20)**	**NC (n = 30)**	**Test’s value**	Statistical result*
Age (years)	59.05 (5.64)	58.43 (3.01)	t = 0.502	*p* = 0.618
Gender	10 F/10 M	17 F/13 M	*x*^*2*^ = 0.214	*p* = 0.686
Formal Education (years)	10.45 (4.86)	11.02 (3.24)	t = −0.62	*p* = 0.515
MMSE (0–30)	26.90 (1.51)	27.46 (1.50)	t = −1.30	*p* = 0.199
Hoehn and Yahr scale (I/II/III)	2.25 (0.63)	-	-	-
Motor UPDRS score	17.05 (3.15)	-	-	-
Duration of disease (years)	5.75 (3.52)	-	-	-
Depression score, BDI	5.80 (2.87)	5.40 (3.71)	t = 0.406	*p* = 0.686
EHI (1–10)	9.55 (0.76)	9.87 (0.73)	t = −0.822	*p* = 0.415

***Note:*** Mean (standard deviations) are given. *n*, Total number of participants; *M*, Male; *F*, Female; *MMSE*, Mini Mental State Examination; *UPDRS*, Unified Parkinson’s Disease Rating Scale; *BDI*, Beck Depression Inventory; *EHS*, Edinburgh Handedness Inventory. *Difference is significant at the *p* < 0.05 level.

### The modeling and classification of emotions

**Figure 1:**
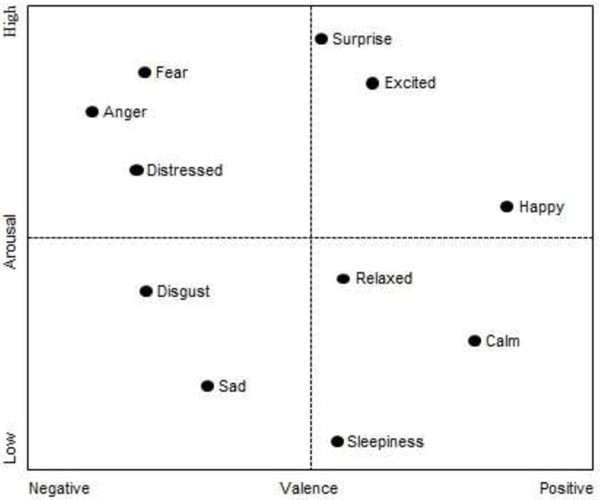
2D emotion model by valence and arousal.

Emotions can be defined as a complex psychophysiological behavior of an individual’s metal state. It is systematically produced by cognitive processes, subjective feelings, physiological arousal, motivational tendencies, and behavioral reactions [38]. In recent years, the emotions have been studied in various fields such as cognitive science, psychology, behavioral science and human computer interaction. Researchers across these disciplines have agreed on two categories of emotional models. The first category includes the discrete emotional model (DEM) where the objective is to recognize the universally accepted six basic emotions namely happiness sadness, fear, anger, disgust and surprise [39]. On the other hand, affective dimensional model (ADM) specifies emotions as a combination of two parameters, namely, valence and arousal [40]. Valence stands for one’s judgment about a situation as positive and negative and arousal spans from calmness to excitement, expressing the degrees of one’s excitation. Figure 1 shows the basic emotions plotted on the 2D valence-arousal plane. In this work, six basic emotions (happiness, sadness, fear, anger, surprise, disgust) based on DEM were considered.

### Stimulus material

Gathering good and meaningful data is essential in any clinical application. In works related to emotion recognition using physiological signal, acquiring emotional data that corresponds to specific emotional state is a challenging one, because of the subjective nature of the emotions and cognitive dependence of physiological signals. This requires the emotional states to be elicited internally in the participants. Until now, most studies on emotion recognition in PD have used only facial stimuli, prosodic stimuli, or both [41,42]. Also, a number of emotion induction techniques using pictures, sounds, music, or multimodal approach (combination of audio & visual) have been used to elicit the target emotions [43-47] in normal controls. Among all these stimuli modality researchers have identified that multimodal stimuli induce emotions in the participants more naturally and effectively than other modalities [45,46,48,49]. In this work, we utilized a multimodal approach to evoke specific targeted emotional state.

The emotional stimuli were taken from different sources such as the International Affective Picture System (IAPS) database [50], International Affective Digitized Sounds (IADS) [51] database and video clips (e.g., funny animals, wonder activities by humans, etc.) collected from various resources on the internet (e.g., YouTube, Facebook and others) [52]. In the work by Brown et al. (2011), the stimuli used to elicit the emotion internally in the participant included both video clips, and combination of pictures & sounds selected from the IAPS and IADS database. The results of the experiment showed that there is no difference when performing the experiment with different sources of stimuli (but same characteristics) to induce emotion internally in the participants [53]. The elicitation of emotions such as sadness, fear, and disgust was mainly attained by using affective pictures from IAPS and sounds from IADS databases. Various psychological and psychophysiological experiments have experienced that these stimuli set has great potential in the investigation of sadness, fear, and disgust emotion [43,53]. Moreover, Mikels et al. [54] & Redondo et al. [55] provided a more complete characterization of the categorical structure of the IAPS and IADS stimulus set, with the objective of identifying images and sounds that elicit one discrete emotion more than other emotions. From this, the IAPS pictures^1^ (disgust: valence mean (SD) = 2.43 (1.51), arousal mean (SD) = 5.90 (2.25); fear: valence mean (SD) = 3.80 (1.89), arousal mean (SD) = 5.85 (2.12); sadness: valence mean (SD) = 2.74 (1.57), arousal mean (SD) =5.00 (2.08)) and IADS sound^2^ (disgust: valence mean (SD) = 4.00 (1.72), arousal mean (SD) = 5.82 (1.93); fear: valence mean (SD) = 4.00 (1.72), arousal mean (SD) = 5.82 (1.93); sadness: valence mean (SD) = 3.28 (1.65), arousal mean (SD) = 6.61 (1.89)) were selected and combined together according to their arousal and valence values provided in the databases. For example, a negative/high aroused sound is matched with a negative/high aroused image. Furthermore, the emotions happiness, surprise, and anger were elicited using video clips. One of the major tasks in inducing emotions using audio-visual clips is to identify video clips that would elicit the target emotions better. For this, around 30 video clips per emotional state were collected from various sources on the internet, and a pilot study was conducted. Thirty volunteers in the mean age of 26.4 years (24 to 45 years) participated in the pilot study to rate the emotions they experienced when watching the video clips. All of them were psychology teachers or students of the UKM medical center, Kuala Lumpur. Thirty audio-visual clips (ten for each emotion) with the highest rating were chosen for data collection.

### Emotion elicitation protocol

**Figure 2:**
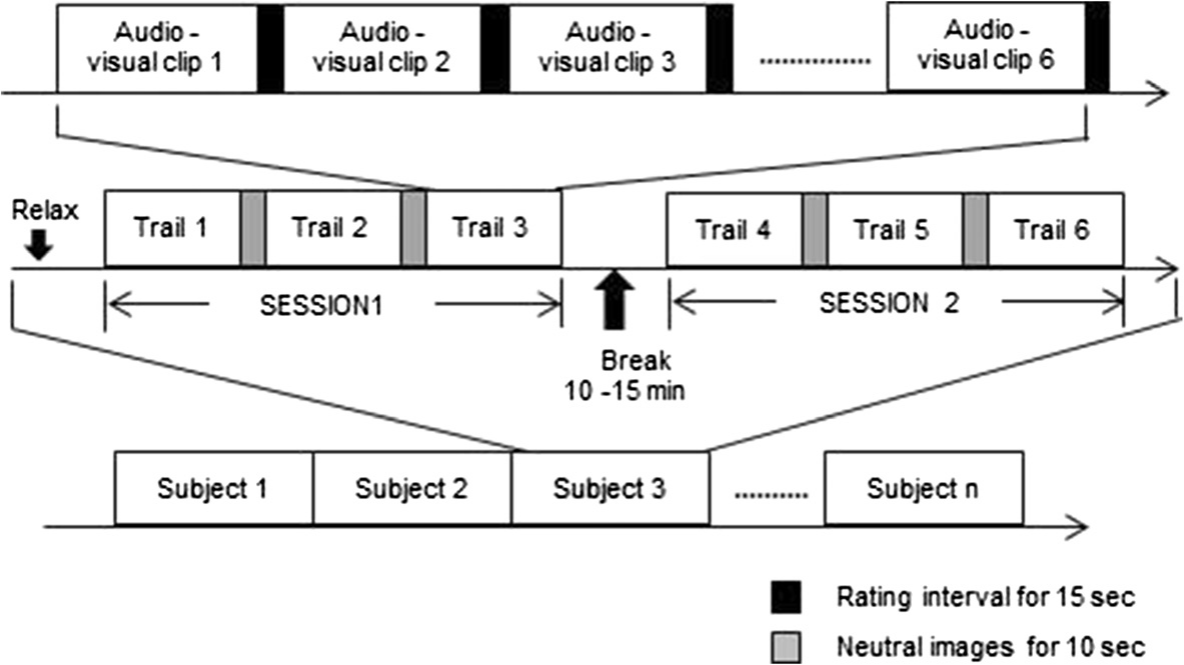
Emotion elicitation protocol.

An illustrated version of the emotion elicitation protocol is shown in Figure 2. As shown, the protocol had two sessions of three trails each. There was a break of 10–15 minutes between the sessions. The participants were allowed to relax during the break (since the continuous assessment would have been too exhausting). The multimodal stimulus relating to all the six emotional states (happiness, sadness, fear, anger, surprise and disgust) was displayed in each trial in a random order. Each combination of picture and sound was presented for six seconds [56]. To maximize the participants’ emotional reactivity, each clip block consisted of six combinations of the same emotional category and lasted for 36-second. In addition, each of the video clips varied from 36–45 seconds in duration, depending on the length of the clip. Neutral images, which can calm down the participant state, were displayed for 10-second at the start of each trail. This would help the participant to get back to the normal or neutral state from emotional excitation. Besides, a 15-second rating interval [57] was provided between the clips in which participants answered on a five point self-assessment scale and also helps to avoid any feedback from the previous emotional stimuli. Each session took about approximately 30 minutes.

### Procedure

**Figure 3:**
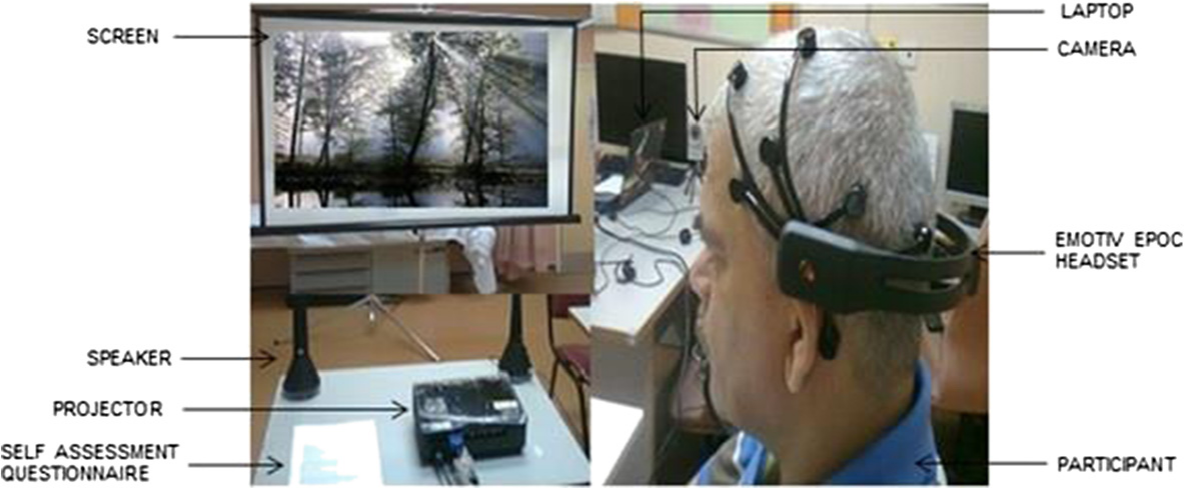
Experimental setup.

The purpose of the study was clearly explained to the participants before starting the experiment. The participants were further requested to relax, minimize their body movement (to reduce the appearance of relevant artifacts in the EEG recordings), and concentrate on the emotional stimuli. Then, self-guided emotion elicitation protocol was displayed on the screen. The experiment set up was shown in Figure 3. At the end of each clip, participants filled a self-assessment questionnaire to state the status and strength of the emotions they felt during the experiment. They were asked to report the strength using a five-point scale according to the degree (1 = very low, 2 = low, 3 = medium, 4 = high, and 5 = very high). The participants were also allowed to indicate multiple emotions during the experiment. A picture of the self-assessment questionnaire is as shown in Table 2.

**Table 2:** Picture of self-assessment questionnaire

**S. NO**	**Audio-visual clip No.**	**Primary emotion experienced**						**Intensity of primary emotion**					**Any other emotion ?**					
		**(√)**						**(√)**					**(√)**					
		**Happiness**	**Sadness**	**Fear**	**Anger**	**Surprise**	**Disgust**	**Very low**	**Low**	**Medium**	**High**	**Very high**	**Happiness**	**Sadness**	**Fear**	**Anger**	**Surprise**	**Disgust**
**1**	**Clip 1**																	
**2**	**Clip 2**																	
**3**	**Clip 3**																	
**4**	**Clip 4**																	
**5**	**Clip 5**																	
**6**	**Clip 6**																	
**7**	**Clip 7**																	
**8**	**Clip 8**																	
**9**	**Clip 9**																	
**10**	**Clip 10**																	

Note: The questionnaire used by the participant to state the status and strength of the emotions they felt during the experiment.

### EEG recording and data analysis

**Figure 4:**
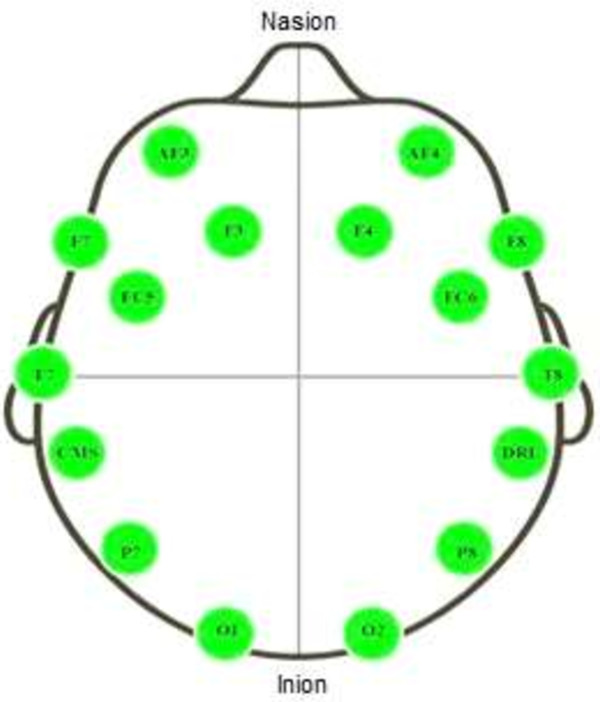
Electrode positions.

EEG recordings were conducted using the Emotive EPOC 14-channel EEG wireless recording headset (Emotive Systems, Inc., San Francisco, CA). The electrode scheme was arranged according to the international 10–20 system and included active electrodes at AF3, F7, F3, FC5, T7, P7, O1, O2, P8, T8, FC6, F4, F8, and AF4 positions, referenced to the common mode sense (CMS-left mastoid)/driven right leg (DRL-right mastoid) ground as shown in Figure 4. The acquired data were digitized using the embedded 16-bit ADC with 128 Hz sampling frequency per channel and sent to the computer through wireless technology. It utilizes a proprietary USB dongle to communicate using the 2.4 GHz band. Prior to use, all felt pads on top of the sensors have to be moistened with a saline solution. In addition, the Emotiv Software Development Kit (SDK) provides packet count functionality to ensure no data is lost. The real time sensor contact quality was visually monitored to ensure quality of measurements.

EEG analysis was performed offline in the MATLAB (version7.12.0.635, R2011a) environment. The raw EEG data was split as per the emotional states. After that, the EEG signals were subjected to filtering. In particular, IIR Butterworth bandpass (6th order filter) was used. The focus was placed upon the four EEG frequency bands: delta (1 – 4 Hz), theta (4 – 8 Hz), alpha (8 – 13 Hz), and beta (13 – 30 Hz) [47,58]. A study published by Kim [56], proposed that the use of different epoch size that depends on modality, e.g., 2–6 seconds for speech, and 3–15 seconds for biosignals. In this study, the EEG signals were segmented into six seconds corresponding to the duration of each multimodal stimuli projection. Then, a separate threshold method was used to remove eye blinking artifacts, in which epochs that were found to have amplitudes exceeding ± 80 μV were excluded from the study [59]. Finally, eighty four artifact-free epochs from middle data segment of each emotional state across delta, theta, alpha, and beta frequency band were selected for further analysis.

The frequency domain analysis was performed using the Fast Fourier Transform (FFT) algorithm (with the resolution of 0.125 Hz) to calculate absolute (μV^2^/Hz) power density, relative (%) power density and mean frequency (Hz) within each of the sub-bands. The absolute power of a band is the integral of all of the power values within its frequency range. Relative power (RP) indices for each band were derived by expressing absolute power in each frequency band as a percent of the absolute power (AP) summed over the four frequency bands. Mean frequency was calculated using a formula published by [60]. Mean (total) frequency (Hz) was also derived from the entire analyzed spectrum (1 – 30 Hz). Measures of inter-hemispheric (absolute) power asymmetry for each band were also computed for seven homologous sites (AF3-AF4, F7-F8, F3-F4, FC5-FC6, T7-T8, P7-P8, O1-O2) and an additional set of ten electrode site pairs (AF3-F3, AF4-F4, F3-O1, F4-O2, FC5-P7, FC6-P8, P7-O1, P8-O2, T7-O1, T8-O2) were used to derive measures of intra-hemispheric power asymmetry for each band as based on the ‘neurometrics’ formulas described by John et al. and Prichep and John [61,62]. Accordingly, right (R) hemisphere vs. left (L) hemisphere asymmetry indices (R-L) were calculated with the formula [(R-L)/(R + L)]. For intra-hemispheric symmetry, anterior (A) (frontal) vs. posterior (P) (back) (A-P) value ratios for each electrode pair were derived with the formula [(A-P)/(A + P)]. Absolut power and asymmetry EEG variables were log transformed (log(χ)) and, relative power variables were transformed by log[χ ÷ (1 ‒ χ)] in order to normalize the distribution of the data [63-65]. As with John et al. [66], the EEG frequency (Hz) indices were found to be normally distributed and thus did not require transformation.

### Statistical analysis

All statistical analyses were performed using the SPSS version 20.0 software package (SPSS Inc., Chicago, IL). The Shapiro-Wilk normality test was used to evaluate whether continuous variables exhibited a normal distribution. Parametric analysis was applied to normal data, whereas nonparametric analysis was applied to non-normal data. A three-way repeated measures (mixed design) analysis of variance (ANOVA) was performed using the factors: Group (PD, normal controls), Emotional states (happiness, sadness, fear, anger, disgust and surprise) and Electrode sites for absolute power, relative power and frequency measures for each frequency band. Similarly, separate ANOVAs were conducted on inter and intra-hemispheric asymmetry measures. The ANOVAs treated Emotional states and Electrode sites as a within subjects factor and Group as between subjects factor. When a main effect of or interactions with Emotional states were found as significantly different between two groups, another ANOVA (two-way) was performed, using only the Emotional states factor values from the selected frequency band. In these analyses Group was the between subjects factor and Electrode sites the repeated factor. Violations of sphericity were adjusted by the Greenhouse- Geiser epsilon correction [67]. When a significant Group x Electrode sites interaction was detected by ANOVA, in order to determine significance of difference for each of the electrodes, a separate two tailed student’s t-test was performed.

In addition, the data from the behavioral study (subjective ratings as well emotion recognition rates) were analyzed separately by repeated ANOVA measures, with group as between-subject factor and emotion as within-subject factor. The results were considered as significant at the level of *p <* 0.05. For all analyses, the uncorrected degrees of freedom and the corrected p-values are reported. Due to space reasons, only significant effects between-group, Emotional state factors and follow up test results are reported here.

## Results

### Behavioral measures

**Figure 5:**
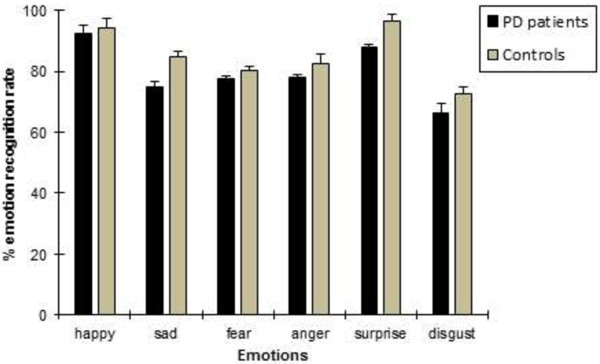
**Means and standard error of means (SEM) of emotion recognition accuracy (%) in PD and NC groups.** Overall, happiness stimuli were recognized best, whereas disgust stimuli were recognized worst

Mean subjective ratings are given in Table 3. As shown, overall the ratings were higher for happiness, and lower for disgust; main effect of emotion [F(5, 240) = 7.88, *p* < 0.0001]. No significant difference between groups and no Group x Emotion interaction were observed (*p* > 0.9). In the emotion recognition task (shown in Figure 5), performance of PD patients did not differ significantly from NC. Overall, happiness emotions were recognized best (% correct M = 93.42; SD = 9.00), whereas disgust emotions were recognized worst (% correct M = 69.58; SD = 3.20), [F(5, 240) = 2.99, *p* = 0.023].

**Table 3:** Mean subjective ratings of emotional stimuli by PD patients and healthy controls

**Emotion**	**PD**		**NC**	
	**Mean**	**SD**	**Mean**	**SD**
Happiness	4.25	1.12	4.37	0.96
Sadness	3.55	1.28	3.77	0.68
Fear	3.45	0.94	3.57	1.07
Anger	3.30	1.17	3.63	0.96
Surprise	3.85	0.75	3.90	1.12
Disgust	2.95	1.10	3.07	1.23

*Note*. Ratings: 1 = very low, 2 = low, 3 = medium, 4 = high, and 5 = very high.

### Absolute/relative power

A three-way repeated measures ANOVA showed significant Emotional state effects were evident with absolute delta [F(2, 332) = 4.124, *p =* 0.017], theta [F(2, 332) = 4.328, *p =* 0.014], alpha [F(2, 332) = 6.332, *p* = 0.002], and beta [F(2, 332) = 4.778, *p* = 0.009] power. Significant Group absolute power differences were limited to theta [F(1, 166) = 29.16, *p* = 0.0001], alpha [F(1, 166) = 20.42, *p =* 0.0001], and beta [F(1, 166) = 8.89, *p* = 0.003] activity. To explain Group x Emotion interaction, a post-hoc two-way ANOVA was performed for each emotion with Group and Electrode pair. This disclosed a significant difference between PD patients and NC group in delta, theta, alpha, and beta frequency band during emotion information processing, with PD patients having less absolute power across all the emotional state. Table 4 shows the summary of absolute power *p*-values obtained from two-way ANOVA. Follow up t-tests showed that PD patients had less absolute power values than in controls at all the scalp sites during emotional stimuli of different categories. In general, the absolute power distributions with theta, alpha, and beta were maximum at anterior sites and delta maximum at posterior sites, bilaterally during the emotional stimuli.

**Table 4:** Summary of significant two way ANOVA p-values for Group x Electrode sites interaction difference between PD patients vs. normal controls with respect to frequency bands

	**Frequency band**	**Emotional states ( *p *-value)**					
		**Happiness**	**Sadness**	**Fear**	**Anger**	**Disgust**	**Surprise**
Absolute Power measures	Delta	0.001	0.028	0.030	0.001	0.002	0.011
	Theta	0.000	0.003	0.004	0.011	0.024	0.016
	Alpha	0.016	0.009	0.004	0.007	0.020	0.008
	Beta	0.006	0.015	0.001	0.005	0.003	0.004
Relative power measures	Delta	0.004	0.001	0.000	0.003	0.003	0.001
	Theta	0.006	0.001	0.001	0.004	0.009	0.006
	Alpha	0.008	0.003	0.001	0.003	0.006	0.004
	Beta	0.006	0.011	0.001	0.002	0.001	0.002

Relative power measures with ANOVAs found that significant Emotional state effects were shown for delta [F(2, 332) = 7.053, *p* = 0.001], theta [F(2, 332) = 3.085, *p =* 0.047], alpha [F(2, 332] = 6.332; p = 0.002), and beta [F(2, 332) = 5.195, *p* = 0.006]. Although significant Group differences were observed with delta [F(1,166) = 18.897, *p* = 0.000], theta [F(1, 166) = 11.265, *p* = 0.001], alpha [F(1, 166) = 46.520, *p* = 0.001] and beta [F(1, 166) = 15.156, *p =* 0.000] activity. Two-way ANOVA on Emotional state values separately confirmed significant influence of Group x Electrode sites interaction in all the bands, which indicated that PD patients show reduced brain electrical activity during the processing of different emotional categories than NC. Table 4 shows the summary of relative power significant difference *p*-values of each emotional categories with respect to frequency bands. The two-tailed t-tests showed that PD patients exhibited significant (*p* < 0.05) differences, with smaller relative power values than normal controls at all scalp sites.

### Hemispheric asymmetry

**Figure 6:**
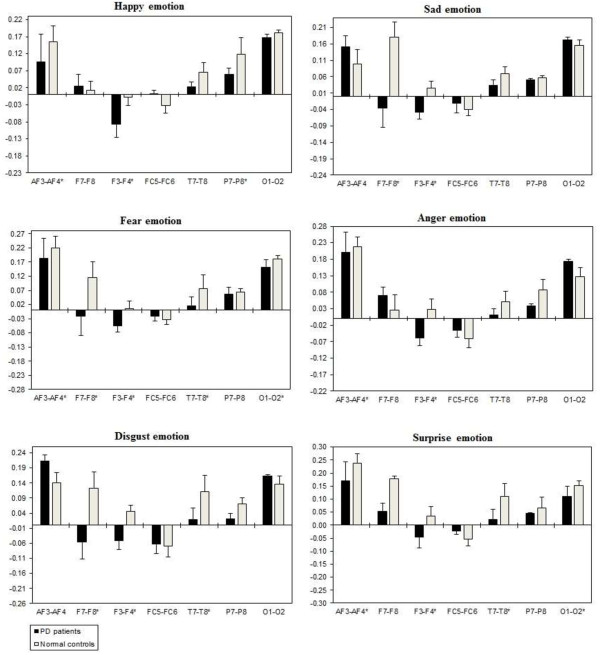
**Inter-hemispheric delta power (in μV**^**2**^**/Hz).** Group mean (± S.E.) log transformed inter-hemispheric delta power asymmetry values (y-axis) for seven site pairs (x-axis) in PD patients and normal controls during emotional stimuli. More negative numbers indicate less delta power. Lower numbers (i.e., more negative) are associated with increased activation [45]. The significant site pair (*p* < 0.05) differences between the groups were marked as *.

**Figure 7:**
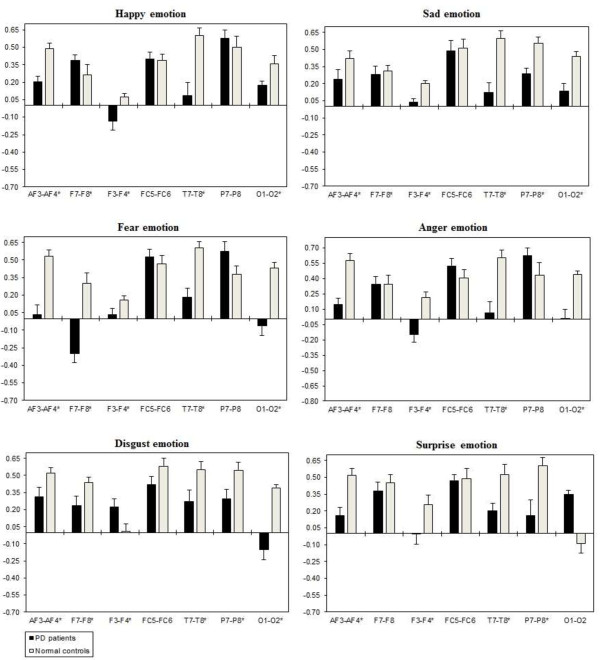
**Inter-hemispheric theta power (In μv**^**2**^**/Hz).** Group mean (± S.E.) log transformed inter-hemispheric theta power asymmetry values (y-axis) for seven site pairs (x-axis) in PD patients and normal controls during emotional stimuli. More negative numbers indicate less delta power. Lower numbers (i.e., more negative) are associated with increased activation [45]. The significant site pair (*p* < 0.05) differences between the groups were marked as *.

**Figure 8:**
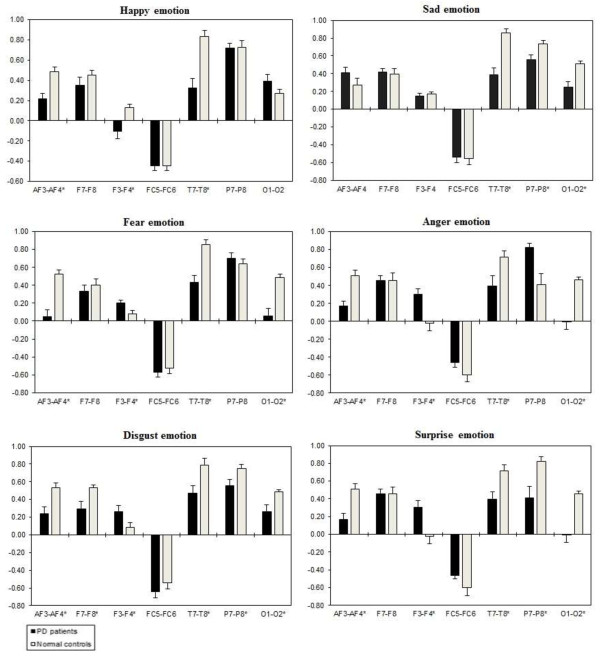
**Inter-hemispheric alpha power (in μV**^**2**^**/Hz).** Group mean (± S.E.) log transformed inter-hemispheric alpha power asymmetry values (y-axis) for seven site pairs (x-axis) in PD patients and normal controls during emotional stimuli. More negative numbers indicate less delta power. Lower numbers (i.e., more negative) are associated with increased activation [45]. The significant site pair (*p* < 0.05) differences between the groups were marked as *.

**Figure 9:**
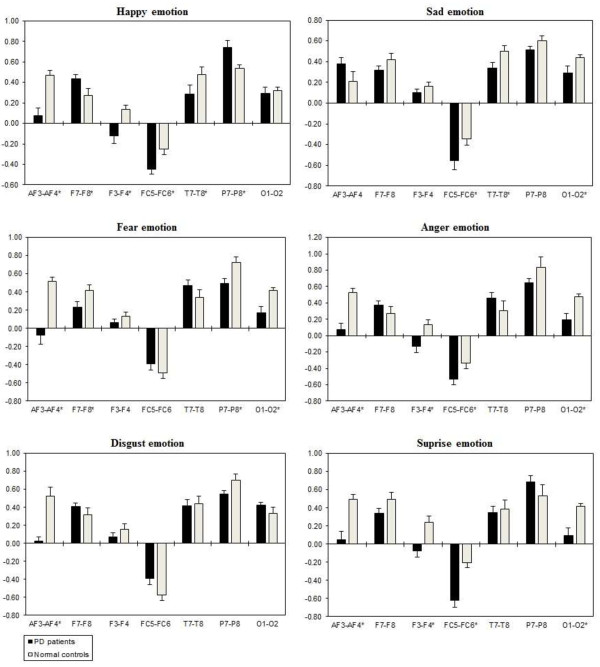
**Inter-hemispheric beta power (in μV**^**2**^**/Hz).** Group mean (± S.E.) log transformed Inter-hemispheric beta power asymmetry values (y-axis) for seven site pairs (x-axis) in PD patients and normal controls during emotional stimuli. More negative numbers indicate less delta power. Lower numbers (i.e., more negative) are associated with increased activation [45]. The significant site pair (*p* < 0.05) differences between the groups were marked as *.

The ANOVA with three way repeated measures interaction revealed significant Emotional state effects for inter-hemispheric delta [F(2, 332) = 3.225, *p* = 0.041], theta [F(2, 332) = 3.225, *p* = 0.014], alpha [F(2, 332) = 3.446, *p =* 0.033], and beta [F(2,332) = 4.253, *p* = 0.015) activity. Two-way ANOVA separately showed a significant Group x Electrode sites interaction with inter-hemispheric delta, theta, alpha, and beta band. This showed that all the Emotional states could be differentiated significantly between the two groups, with less band power (i.e., reduced brain activity) in the PD group when compared to the normal control group. Table 5 shows the summary of inter-hemispheric significant difference *p*-values of each emotional categories with respect to frequency bands. Figures 6, 7, 8 and 9 summarize the mean ± Standard error (S.E) significance of differences between PD patients and NC group in delta, theta, alpha, and beta band for each of the Electrode sites, as revealed by an independent t-tests. Significant Group inter-hemispheric differences were limited to theta [F(1, 166) = 20.802, *p =* 0.0001], alpha [F(1, 166) = 46.612, *p* = 0.0001], and beta [F(1,166) = 9.152, *p =* 0.003). In general, inter-hemispheric theta, alpha and beta ratio values were smaller at anterior regions across the significant electrode pairs. Whereas both groups evidenced positive asymmetry ratio values during the emotional stimuli indicating greater right than left hemisphere power, the positive values were generally larger in the normal controls than in the PD patients.

**Table 5:** **Summary of significant two way ANOVA *p*-values for Group x Electrode sites interaction difference between PD patients vs. normal controls with respect to frequency bands**

	**Frequency band**	**Emotional states** (***p*-values)**					
		**Happiness**	**Sadness**	**Fear**	**Anger**	**Disgust**	**Surprise**
Inter-hemispheric power measures	Delta	0.005	0.008	0.000	0.001	0.002	0.007
	Theta	0.006	0.001	0.003	0.004	0.024	0.011
	Alpha	0.005	0.025	0.002	0.002	0.035	0.010
	Beta	0.004	0.018	0.002	0.016	0.018	0.007
Intra-hemispheric power measures	Delta	0.015	0.016	0.005	0.016	0.003	0.001
	Theta	0.006	0.001	0.000	0.002	0.007	0.004
	Alpha	0.009	0.002	0.000	0.002	0.005	0.002
	Beta	0.006	0.005	0.000	0.001	0.001	0.000

**Figure 10:**
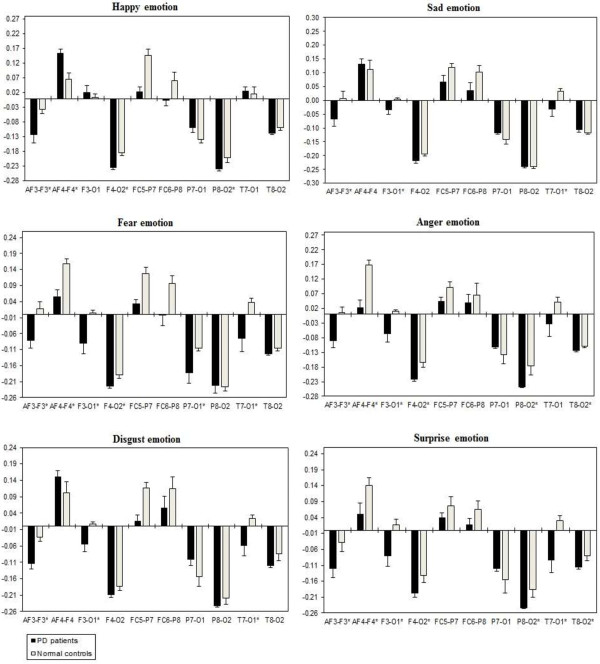
**Intra-hemispheric delta power (in μV**^**2**^**/Hz).** Group mean (± S.E.) log transformed Intra-hemispheric delta power (in μV^2^/Hz) asymmetry values (y-axis) for seven site pairs (x-axis) in PD patients and normal controls during emotional stimuli. More negative numbers indicate less delta power. Lower numbers (i.e., more negative) are associated with increased activation [45]. The significant site pair (*p* < 0.05) differences between the groups were marked as *.

**Figure 11:**
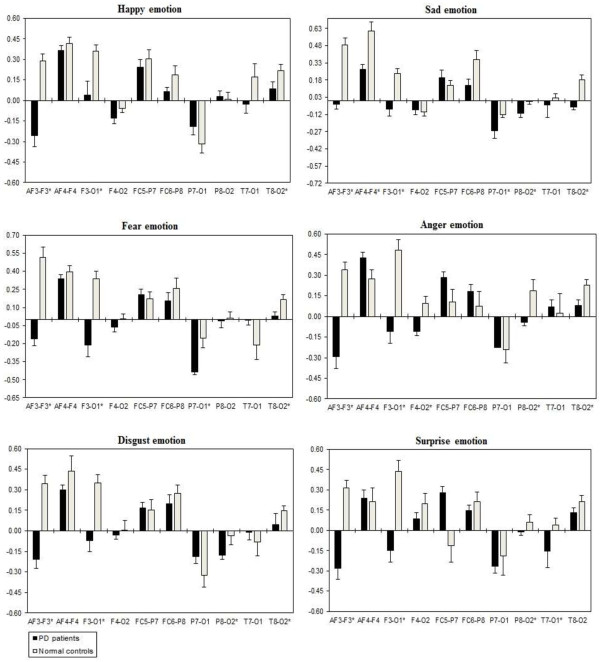
**Intra-hemispheric theta power (in μV**^**2**^**/Hz).** Group mean (± S.E.) log transformed intra-hemispheric theta power asymmetry values (y-axis) for seven site pairs (x-axis) in PD patients and normal controls during emotional stimuli. More negative numbers indicate less delta power. Lower numbers (i.e., more negative) are associated with increased activation [45]. The significant site pair (*p* < 0.05) differences between the groups were marked as *.

**Figure 12:**
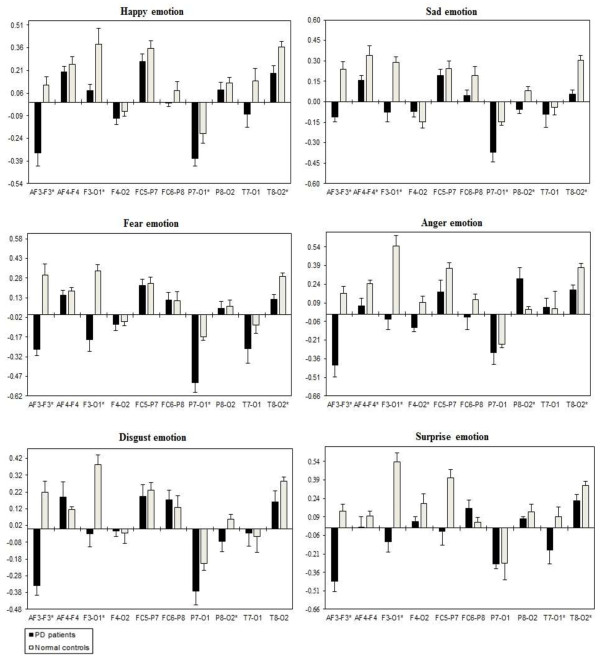
**Intra-hemispheric alpha power (in μV**^**2**^**/Hz).** Group mean (± S.E.) log transformed intra-hemispheric alpha power (in μV^2^/Hz) asymmetry values (y-axis) for seven site pairs (x-axis) in PD patients and normal controls during emotional stimuli. More negative numbers indicate less delta power. Lower numbers (i.e., more negative) are associated with increased activation [45]. The significant site pair (*p* < 0.05) differences between the groups were marked as *.

**Figure 13:**
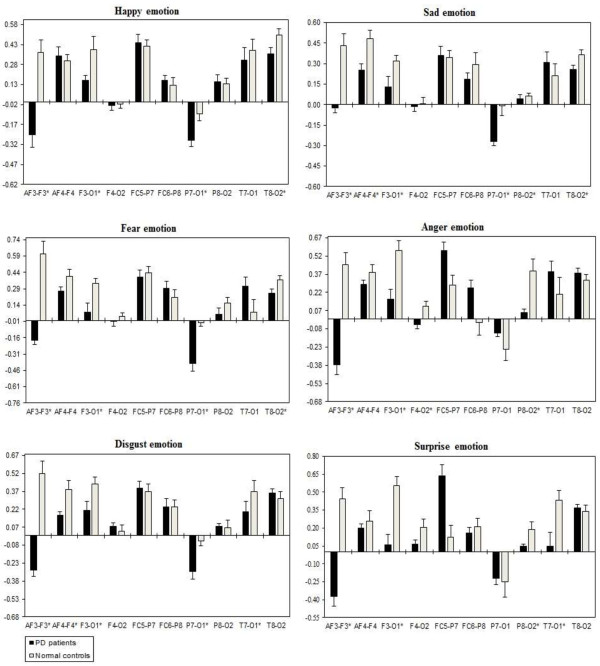
**Intra-hemispheric beta power (in μV**^**2**^**/Hz).** Group mean (± S.E.) log transformed intra-hemispheric beta power asymmetry values (y-axis) for seven site pairs (x-axis) in PD patients and normal controls during emotional stimuli. More negative numbers indicate less delta power. Lower numbers (i.e., more negative) are associated with increased activation [45]. The significant site pair (*p* < 0.05) differences between the groups were marked as *.

A three way repeated ANOVA measures found significant Emotional states were shown for intra-hemispheric delta [F(5,332) = 3.416, *p* = 0.034], theta [F(5,332) = 3.153, *p* = 0.044], alpha [F(5,332) = 3.107, *p =* 0.046], and beta [F(5,332) = 3.225, *p =* 0.041]. Two ways ANOVA on Emotional state values separately confirmed significant influence of Group x Electrode sites interaction with intra-hemispheric delta, theta, alpha, and beta, with decreased band power (i.e., less emotional activity) in PD patients than normal controls. Table 5 shows the summary of intra-hemispheric significant difference *p*-values of each emotional category with respect to frequency bands. Figures 10, 11, 12 and 13 summarize the mean (± S.E.) significance of differences between PD patients and NC group in delta, theta, alpha, and beta band for each of the Electrode sites, as revealed by independent t-test. In general, intra-hemispheric ratio values were smallest with F4-O2 and P7-O1 and largest with AF4-F4, and F3-O1 site pairings with respect to normal controls. Although significant Group intra-hemispheric differences were limited to alpha [F(1,166) = 6.613, *p* = 0.011], with normal controls exhibiting greater ratio values (indicating relatively greater alpha at anterior vs. posterior sites of each pair) than patients during emotion processing. Follow-up tests found the two groups to differ with respect to significant site pairs in both right and left hemisphere (i.e., bilaterally). For the significant site pairs, both groups exhibited both negative/positive ratio values, indicating evenly distributed power across anterior and posterior electrodes of these site pairs, but values were less in the patients, than in the normal controls during emotional stimuli.

### Mean frequency

**Figure 14:**
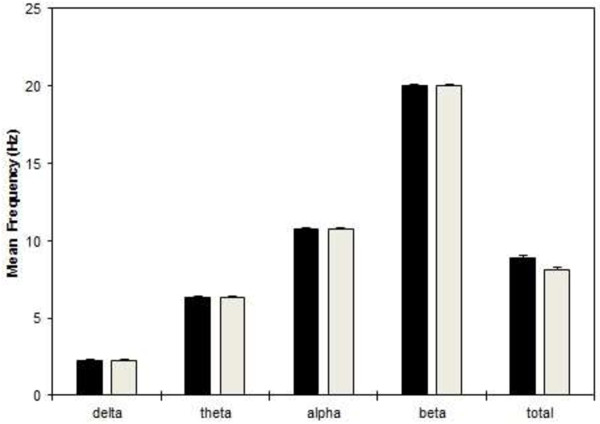
**Mean frequencies during emotion processing.** Group mean (± S.E.) non-log transformed frequency (in Hz) values, collapsed across all sites during emotional stimuli for delta, theta, alpha, beta and total spectrum bands in PD patients and normal controls

A three way repeated ANOVA measures did not find any significant Emotional state effects with delta (*p* = 0.564), theta (*p* = 0.280), alpha (*p =* 0.407), beta (*p* = 0.236) and total mean frequency (*p* = 0.163) during emotional stimuli. Mean (± S.E.) delta, theta, alpha, beta band and total spectrum frequency values for each group collapsed across Emotional states and Electrode sites are shown in Figure 14. In general, delta frequency values were higher at frontal-central sites, and theta frequency values were smaller at temporal and occipital sites. Alpha and beta frequencies values were higher at posterior sites, and mean total frequency value was higher at anterior sites. Although no significant group differences were observed for mean delta, theta, alpha and beta frequency, significant Group [F(1, 166) = 4.522, *p* = 0.034] effects were found for mean total frequency. Total frequency was generally higher in patients, reaching significance (*p* < 0.05) at six anterior (AF3, F7, F3, F4, F8, and AF4) of the fourteen targeted electrode sites during emotion information processing.

### Discriminant classification

With respect to discriminant analysis, we only used the indices (by three way repeated ANOVA measures) that differentiated PD patients and normal control groups during emotion information processing. To further reduce the number of predictors, values within significant band index were averaged across the significant sites/site pairs which showed patients and controls to be different, thus resulting in one value per band for each index. The statistical procedure used was multiple stepwise discriminant analysis. Table 6 displays the results of the initial discriminant and the independent replication (cross-validation) classification accuracy from the separation of PD patients from NC group based on EEG variables collapsed across all the emotional categories. The overall classification was 95.0% in both classification attempts. Theta, alpha, and beta absolute power; delta, theta, alpha, and beta relative power; theta, alpha, and beta inter-hemispheric power asymmetry; and alpha intra-hemispheric power asymmetry contributed as features to the classification. The independent replication demonstrates the high replicabilty and stability of this discriminant function.

**Table 6:** Classification of PD patients and normal controls based on discriminant analysis of EEG variables collapsed across all emotional categories

**Actual group**	**n**	**% Correct**	**Classifications**		**% Correct**	**Classifications**	
			**I**	**II**		**I**	**II**
			**Initial discriminant**			**Independent replication**	
I. PD patients	20	100.0	20	0	100.0	20	0
II. Normal controls	30	90.0	3	27	90.0	3	27
Total%		95.0			95.0		

## Discussion

The present study is to our knowledge the first to examine a relatively wide range of spectrally and statistically derived EEG features in relation to emotion information processing in PD patients. Significant patient vs. control group differences were seen with a number of features during emotion information processing. In line with previous findings on behavioral measures [5,16,17,21], the present study also found PD patients to report no impairment in emotion recognition accuracy, and subjective ratings of emotional stimuli. It is noteworthy that these findings are most likely not due to low statistical power since PD patients were descriptively even better in recognizing emotion disgust and fear compared to normal controls. Moreover, Cohen et al. [68] found that PD patients under dopamine replacement therapy (DRT) were not impaired in emotion recognition, but were more sensitive to cognitive load, which seems to be especially true for non-demented and non-depressive participants as in our study. On the other hand, absolute and relative power revealed that PD patients showed lower power values than normal controls during the processing of happiness, sadness, fear, anger, surprise, and disgust emotions. Significant group differences between PD patients and normal controls were limited to theta, alpha and beta frequency bands. These findings indicate the neuropathological evidence that PD could be associated with the slowing of oscillatory brain activity [69,70]. This slowing of brain activity exhibits a significant correlation with progression of Hoehn and Yahr stage in PD [71]. Although our PD samples were tested on dopaminergic medication, they still revealed signs of dopamine deficiency as indicated by a mean value of 17.05 in the motor part of the UPDRS. In addition, some of the medications are known to be associated with impulse control disorders in PD, as these aspects might have implications for emotion processing in patient population [72-74].

With respect to inter-hemispheric EEG, PD patients tended to show positive interhemispheric theta, alpha, and beta ratios, indicating relative right hemisphere hypoactivation, but this was more evidenced in NCs and was not limited to anterior sites as group differences were seen at almost all homologous site pairs. This was the result of power asymmetry ratio analysis, with patients exhibiting relatively less right frontal activation. The present inter-hemispheric results support the previously reported traditional theories of emotional processing suggesting right hemisphere specialization for the perception and recognition of social cues [75]. More recently, lateralization of emotion recognition in PD has been debated. For example, Clark et al. reported no asymmetry effects on explicit emotion categorization [8]. Ariatti et al. and Yip et al. reported problems in categorizing disgust prosody in patients with predominantly right-sided [23,24]. While Ventura et al. reported that predominantly left sided patient’s exhibit recognition of sadness emotion [25]. Intra-hemispheric EEG showed significant alpha band group differences, with evenly distributed power across anterior and posterior electrodes, with patients exhibited reduced intra-hemispheric values at all of the significant site pairs. Anteriorization was also evident for mean total spectrum frequency, with mean frequency being higher in patients at bilateral pre-frontal, frontal and central sites. The utility of EEG as a clinical tool in the diagnosis of psychiatric disorders is progressing [John, 1989], its routine use in clinical practice remains in doubt until appropriate investigations are carried out on the reliability, sensitivity, and specificity of these tests [Nuwer 1988]. However, the current discriminant analysis, carried out retrospectively in a non-blinded fashion, did reveal a marked separation of PD patients and controls, yielding an overall accuracy of 95.0%, correctly classifying 27/30 normal controls (90.0% specificity) and 20/20 PD patients (100% sensitivity) in the separation of PD patients and normal controls during emotion information processing. This discriminant analysis utilized power measures and asymmetry indices as features.

Furthermore, the amygdala’s involvement in emotional processing is now well documented in the literature [76,77]. Interestingly, neuropathological research findings support the theory of amygdala impairment in PD. For example, Tessitore et al. investigated the activation of the amygdala in PD patients during emotion processing using fMRI, and found that PD patients exhibited weaker amygdala activation in response to emotional stimuli than NC [78]. Similarly, absence of amygdala response in PD patients was also found by Yoshimura et al. [79]. Additionally, Cancelliere and Kertz (1990) reported that patients with cortical lesions who had additional damage to the basal ganglia showed the most evident deficits in emotion information processing [80]. There is also a large body of evidence pointing to the involvement of dopamine in emotional process [81]. For example, the association between dopaminergic activity and emotion processing has been demonstrated in healthy male volunteers that received dopamine D2-antagonist, which caused an impaired recognition of angry faces [82]. Sprengelmeyer et al. investigated the effect of dopamine medication and observed impaired emotion information processing [10]. This deficit was more severe in non-medicated patients than in medicated patients with PD. Using PET, it has been shown that reduced dopamine transporter (DAT) availability was related to decrease in activation of emotional gesture recognition [83]. Similarly, an fMRI study revealed that the activity of several limbic regions (amygdala, hippocampus, anterior cingulate cortex) during the perception of unpleasant images was reduced in normal controls that had been given a dopaminergic antagonist [84]. These results have been confirmed by other fMRI studies using dopamine manipulations [85].

To sum up, the current study revealed that PD showed no impairments in the behavioral measures, but exhibited deficits in emotional information processing as reflected in neurophysiological measures. This indicated that distributed spectral power in different frequency bands might provide meaningful information about emotional processes in PD patients. Further controlled studies with PD patients ON and OFF medication could help to clarify the influence of the dopaminergic medication on emotion processing. In general, PD is a complex neurodegenerative disease; with significant influences on brain activity. Therefore, as a step forward, it is necessary to apply new emotion recognition analysis methods to extract more typical features from EEG signals of PD patients, and further make classification analysis based on those characteristics indices, which may have potential use as biomarkers of PD and provide an objective technique for the investigation of emotional state changes in PD.

## Appendix

^1^The following pictures were used for emotion induction: **Disgust:** 1945, 2352.2, 3000, 3010, 3015, 3030, 3051, 3060, 3061, 3071, 3080, 3110, 3120, 3130, 3140, 3150,3160, 3250, 3400, 7360, 7361, 7380, 8230, 9040, 9042, 9181, 9290, 9300, 9320, 9330, 9373, 9390, 9405, 9490, 9570, 9830. **Fear:** 1019, 1022, 1030, 1040, 1050, 1051, 1052, 1070, 1080, 1090, 1110, 1111, 1113, 1120, 1200, 1201, 1220, 1230, 1240, 1280, 1274, 1300, 1301, 1302, 1321, 1390, 1930, 1931, 3280, 5970, 5971, 5972, 6370, 9584, 9594, 9592. **Sadness:** 2205, 2271, 2276, 2490, 2520, 2590, 2700, 2800, 2900, 3220, 3230, 3300, 3301, 3350, 6570, 6838, 8010, 9000, 9041, 9050, 9120, 9190, 9210, 9220, 9331, 9410, 9415, 9470, 9520, 9530, 9561,9611, 9910, 9911, 9920, 9921.

^2^The following sounds were used for emotion induction: **Disgust:** 134, 115, 251, 262, 284, 698, 702, 711, 712, 713, 714, 720, 728, 729, 730, 732, 812, 813. **Fear:** 106, 133, 170, 171, 275, 276, 277, 279, 291, 312, 378, 380, 424, 425, 500, 626, 627, 699, 817. **Sadness**: 115, 150, 260, 261, 278, 280, 285, 286,290, 293, 295, 310, 311, 368, 403, 420, 422, 501, 600, 625.

## Abbreviations

PD: Parkinson’s disease; EEG: Electroencephalogram; NC: Normal controls; HUKM: Hospital University Kebangsaan Malaysia; H & Y: Hoehn & Yohr; UPDRS: Unified Parkinson’s disease Rating Scale; MMSE: Mini Mental State Examination; BDI: Beck Depression Inventory; DEM: Discrete Emotional Models; ADM: Affective Dimensional Models; IAPS: International Affective Picture system; IADS: International Affective Digitized Sounds; FFT: Fast Fourier Transform; ANOVA: Analysis of Variance; DRT: Dopamine Replacement Therapy.

## Competing interest

The authors declare that they have no competing interests.

## Authors’ contributions

RY carried out the data acquisition and analysis, participated in the sequence alignment and drafted the manuscript. MM and KS conceived the study, and participated in its design and coordination and helped to draft the manuscript. NMI, KM and MS helped in the PD patients and healthy control participant recruitment. ME helped to carry out the statistical analysis. Finally, RP helped in the language revision of the manuscript. All authors read and approved the final manuscript.
